# Enhanced Thermostability of D-Psicose 3-Epimerase from *Clostridium bolteae* through Rational Design and Engineering of New Disulfide Bridges

**DOI:** 10.3390/ijms221810007

**Published:** 2021-09-16

**Authors:** Jingyi Zhao, Jing Chen, Huiyi Wang, Yan Guo, Kai Li, Jidong Liu

**Affiliations:** 1 College of Light Industry and Food Engineering, Guangxi University, Nanning 530004, China; zhaojy@st.gxu.edu.cn (J.Z.); chenjing@st.gxu.edu.cn (J.C.); Whyi@st.gxu.edu.cn (H.W.); GuoYan@st.gxu.edu.cn (Y.G.); gxlikai@gxu.edu.cn (K.L.); 2 Sugar Industry Collaborative Innovation Center, Guangxi University, Nanning 530004, China

**Keywords:** disulfide bonds, cysteine, molecular dynamics simulation, cold-shock

## Abstract

D-psicose 3-epimerase (DPEase) catalyzes the isomerization of D-fructose to D-psicose (aka D-allulose, a low-calorie sweetener), but its industrial application has been restricted by the poor thermostability of the naturally available enzymes. Computational rational design of disulfide bridges was used to select potential sites in the protein structure of DPEase from *Clostridium bolteae* to engineer new disulfide bridges. Three mutants were engineered successfully with new disulfide bridges in different locations, increasing their optimum catalytic temperature from 55 to 65 °C, greatly improving their thermal stability and extending their half-lives (t_1/2_) at 55 °C from 0.37 h to 4−4.5 h, thereby greatly enhancing their potential for industrial application. Molecular dynamics simulation and spatial configuration analysis revealed that introduction of a disulfide bridge modified the protein hydrogen–bond network, rigidified both the local and overall structures of the mutants and decreased the entropy of unfolded protein, thereby enhancing the thermostability of DPEase.

## 1. Introduction

D-Psicose, or D-allulose, a C-3 epimer of D-fructose, is a sugar substitute with a ~70% sweetness index relative to sucrose, but almost zero calories [1]. In April 2019, the US Food and Drug Administration (FDA) announced that D-allulose was to be excluded from the total and added sugar contents on nutrition labels when used as an ingredient, thereby greatly expanding its potential market. An efficient enzymic synthesis of D-allulose by D-psicose 3-epimerase (DPEase) catalysis has been attracting strong industrial interest [2]. However, chemical manufacturing and biosynthetic processes generally occur under harsh conditions, so a cost-effective biocatalytic manufacturing process for D-allulose is not feasible because of the poor thermostability of natural DPEases [3]. Therefore, developing thermostable DPEase mutants is an urgent requirement for industrial manufacture of D-allulose.

Directed evolution based on random mutagenesis and high-throughput screening approaches is a general strategy for improving enzyme thermostability [4]. This effective method has been successfully applied to, for example *Pseudomonas fluorescens* lipase, *Agrobacterium tumefaciens* uronate dehydrogenase and *Bacillus clausii* alkaline protease [5,6,7]. Nevertheless, directed evolution involves multiple rounds of mutations and creating a large mutant library, which is a labor- and time-consuming process [8]. However, bioinformatics tool development and improved understanding of the factors controlling enzyme thermostability now allow the design of thermostability improvements by computer simulation [9]. Although site-directed mutagenesis has been applied to increase the half-life of *Clostridium bolteae* DPEase by 1.67-fold, the thermostability and sequence homology of DPEases identified so far are generally low, resulting in difficulty selecting templates for multiple sequence alignment [10]. Salt bridges that strengthen intramolecular electrostatic interactions can contribute to improved enzyme thermostability; introduction of salt bridges yielded a 1.89-fold increase in residual activity of a mutant 1,4-α-glucan branching enzyme, compared with the wild-type after treatment at 60 °C for 2 h [11]. However, rational design of salt bridges by statistical analysis of structural information remains challenging because of the incomplete understanding of the salt bridge formation rules [12].

Directed introduction of disulfide bridges into a target protein is regarded as an effective approach for enhancing enzyme thermostability among computational design strategies because disulfide bridges are structurally well-defined and easily characterized [13]. Disulfide bridge(s) formed by the oxidation of two nonadjacent cysteines could stabilize the three-dimensional (3D) protein tertiary structure through reducing the configurational entropy of the unfolded state [14]. With the development of the interdisciplinary approach, the thermostability of various enzymes, such as xylanase, lipase and β-xylosidase, has been improved by engineering additional disulfide bridges [15,16,17]. The selection of appropriate sites for cysteine residue pairs remains challenging, however, because extra disulfide bridges sometimes make no difference to, or decrease, the thermostability of the enzyme [18]. Furthermore, disulfide scrambling sometimes occurs, thereby accelerating the formation of inclusion bodies in the producing organism and affecting the expression of soluble proteins [19].

There have been several developments designed to improve recombinant protein production, especially correct folding and disulfide bridge formation. The *Escherichia coli* Rosetta-gami B (DE3) producer-strain was designed with many modifications to improve the activity and solubility of recombinant proteins, including enhancement of correct disulfide bridge formation, through mutations to improve glutathione reductase and thioredoxin reductase [20]. Unlike the commonly used pET series plasmids, pCold was designed as a cold-shock vector that includes a translation-enhancing element, a highly efficient Shine–Dalgarno sequence (GAGG) and a trigger factor (TF) chaperone, which facilitates co-translational folding of newly expressed polypeptides and high-yield protein expression [21].

The aim of this study was to obtain thermostable mutants of DPEase within a limited screening library of disulfide bridges, via rational design and the use of the improved techniques described above. It provides potential industrial DPEase biocatalysts and detailed theoretical information for the commercial-scale manufacture of D-allulose.

## 2. Results

### 2.1. Computational Design and Screening of Disulfide Bonds

**Figure 1:**
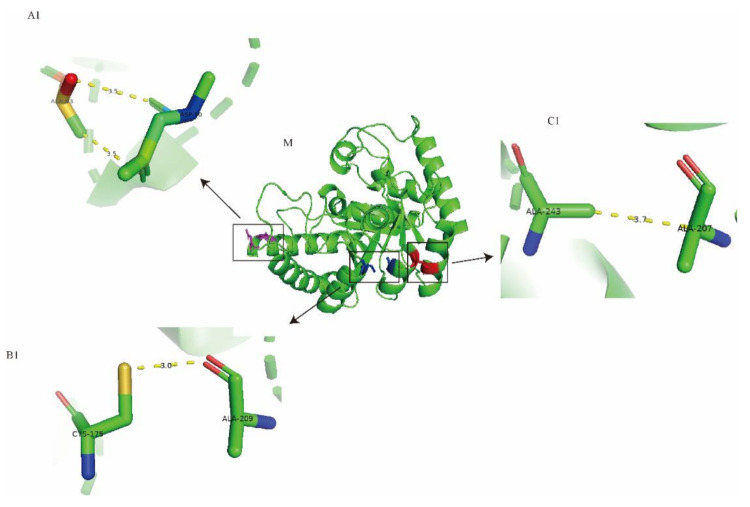
The three-dimensional (3D) model of wild type (M) and three potential mutation pairs, D90/A93 (A1), C175/A209 (B1) and A207/A243 (C1). The 3D molecular visualization was performed with PyMOL software.

To explore the structural location of potential mutation sites, the tertiary structure of Cb-DPEase was constructed with *A. tumefaciens* DPEase as the template (Figure 1). As a prerequisite for conducting mutation analysis, the quality of the Cb-DPEase simulation model was confirmed. The Ramachandran plot produced for the refined model of Cb-DPEase showed 93.4% of residues in favorable regions, 6% in allowed regions and only 0.6% in disallowed regions, indicating that the backbone dihedral angles ψ (psi) and φ (phi), in the predicted model were accurate (Figure S1). Furthermore, the quality of fit for each amino acid sequence to the environment dictated by the protein conformation, was evaluated using Verify3D. For Cb-DPEase, 95.9% of the residues had an average 3D–1D score ≥ 0.2, indicating good compatibility between the atomic model (3D) and the target amino acid sequence (1D).

The DbD computational tool was used to predict candidate residue pairs suitable for substitution by cysteine, to form extra disulfide bridges (Table A2). To quantify the flexibility of the protein, B-factor values were introduced to describe the conformational state of sections of the protein structure. The higher the B-factor value, the more flexible the conformation of the corresponding sections [22]. Three mutation pairs (D90-A93, C175-A209 and A207-A243) with potential to form thermostability-enhancing disulfide bridges were selected because of their high B-factor values, corresponding to the most obvious degrees of thermal motion.

### 2.2. Determination of Disulfide Bridge Content in Purified Mutant Enzymes

**Figure 2:**
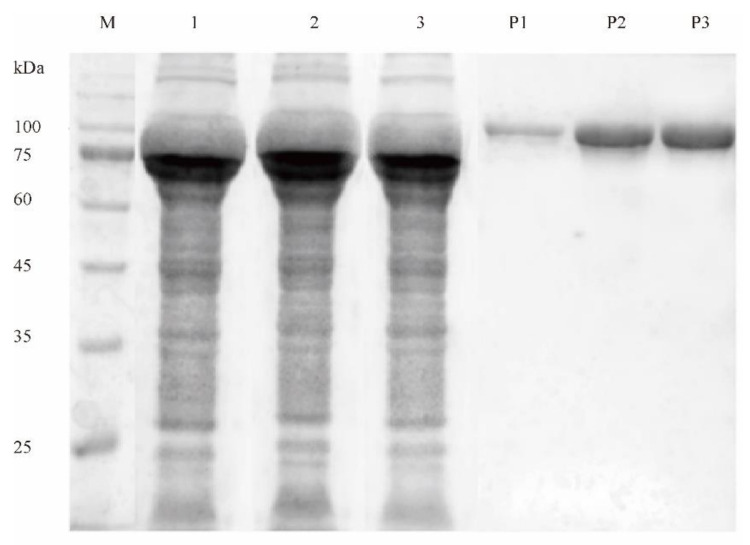
SDS-PAGE analysis of the purified DPEases. M, protein marker; lane 1–3, supernatant of D90C-A93C, C175-A209C, A207C-A243C; lane P1–P3, purified D90C-A93C, C175-A209C, A207C-A243C.

To obtain the purified protein fraction, the wild-type DPEase and its mutants were fused with a C-terminal 6-histidine-tag, followed by purification with Ni^2+^-chelating affinity columns. The molecular masses of D90C-A93C, C175-A209C, and A207C-A243C measured by SDS-PAGE were all about 88.5 kDa, consistent with the theoretical molecular weight (Figure 2).

To determine whether the introduced cysteines were free, or had formed disulfide bridges, the number of free cysteines within the mutant enzymes was measured, compared with those of the wild-type Cb-DPEase. Although two extra cysteine residues each had been introduced into the mutants D90C-A93C and A207C-A243C, the number of free thiol groups did not increase compared with wild-type Cb-DPEase (Table 1). The number of free thiols decreased from five to four in C175-A209C, because the amino acid at position 175 is already a cysteine. These results indicated that new disulfide bonds had formed successfully in these mutants.

### 2.3. Enzymatic Characterizations of Wild-Type Cb-DPEase and the Mutants

**Figure 3:**
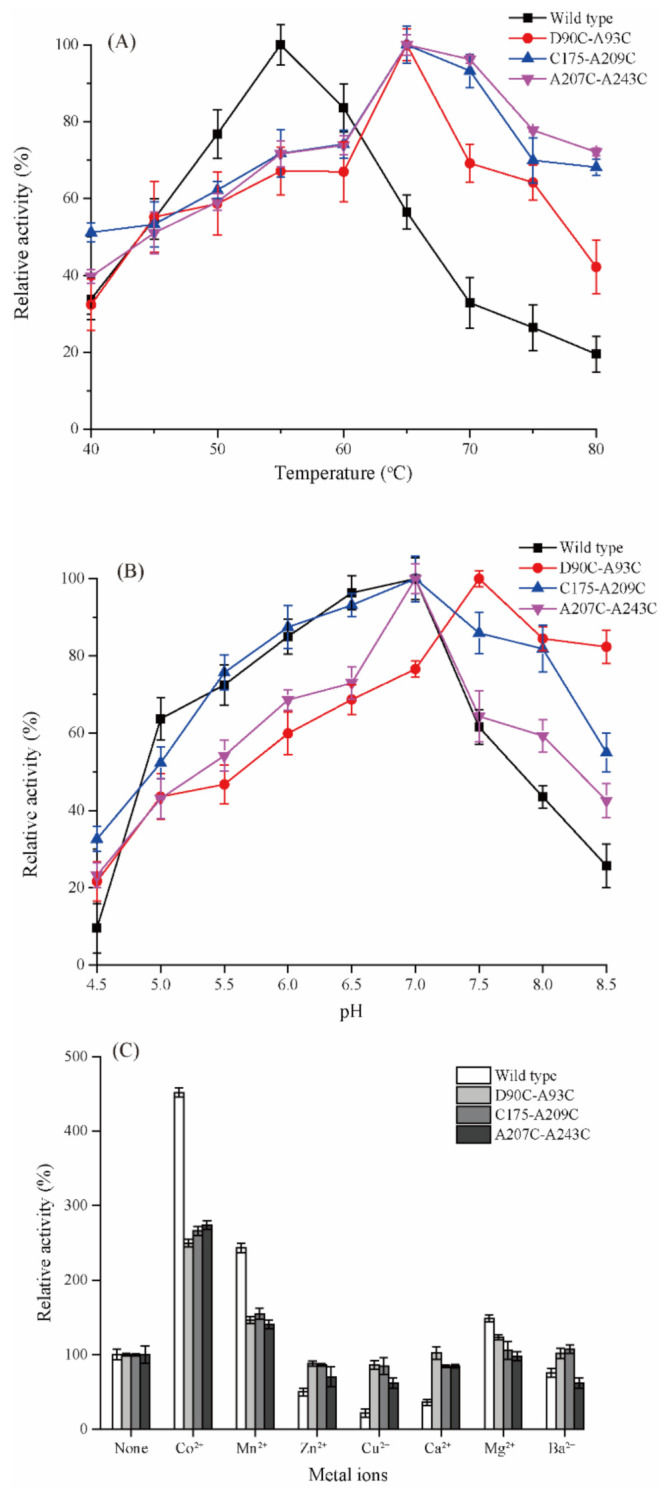
The optimal temperature (**A**), optimal pH (**B**), and effect of metal ions (**C**) of wild type DPEase and the three mutants.

According to the temperature preference assay, the optimum temperatures of all three mutants increased from 55 to 65 °C, while retaining >80% of their relative activities at temperatures between 65 and 75 °C (Figure 3A), meaning that the higher temperatures were necessary to activate the full catalytic activities of the mutants.

The optimum pH of D90C-A93C shifted from 7.0 to 7.5, after introduction of the disulfide bridge and it was more stable at pH 8.0 than the other two mutants, retaining 81% of its activity. C175-A209C and A207C-A243C had the same optimal pH values (7.0) as the wild type; the relative activity of C175-A209C was more than 80% in the pH range 6.0–7.5 (Figure 3B).

The presence of Co^2+^ strongly activated all four enzymes; the activities of wild type, D90C-A93C, C175-A209C and A207C-A243C with Co^2+^ were 3.1-, 2.4-, 2.1-, and 2.0-fold higher than control, reached to 16.03 ± 0.96, 11.95 ± 0.67, 11.03 ± 0.87 and 10.98 ± 0.73 U/mg, respectively (Figure 3C). Mn^2+^ had a weaker activating effect, whereas Cu^2+^ and Zn^2+^ reduced the activities of the mutant enzymes below wild-type.

### 2.4. Effect of Introduced Disulfide Bonds on Thermostability

**Figure 4:**
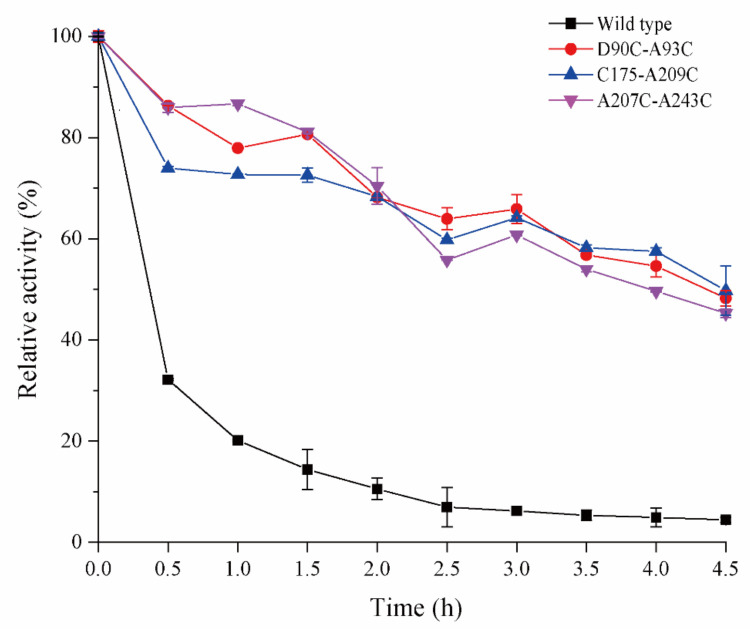
Thermostability of wild type DPEase and the three mutants.

To evaluate the thermal stability of the three mutant enzymes, the half-life (*t*_1/2_) was measured for the wild-type Cb-DPEase and its mutants. Each of the three introduced disulfide bridges resulted in a substantial increase in DPEase thermostability. The half-lives (*t*_1/2_) of D90C-A93C, C175-A209C and A207C-A243C at 55 °C increased to ~4.4 h, ~4.5 h and ~4.0 h, respectively, far higher than the 0.37 h for the wild-type (Figure 4). C175-A209C had the highest residual activity after 4.5 h, with that of D90C-A93C slightly lower. Overall, the three stabilized mutants had markedly better catalytic performance than the wild-type Cb-DPEase at higher temperatures. As shown in Table 2, the observed thermostabilities of D90C-A93C, C175-A209C and A207C-A243C outperforms most other sources of DPEase. Although the three mutations had lower thermostabilities than *Clostridium cellulolyticum* H10 DPEase, optimal pH was found to be slightly lower, which portends its good industrial applicability.

### 2.5. MD Simulation of Wild-Type Cb-DPEase and the Mutants

**Figure 5:**
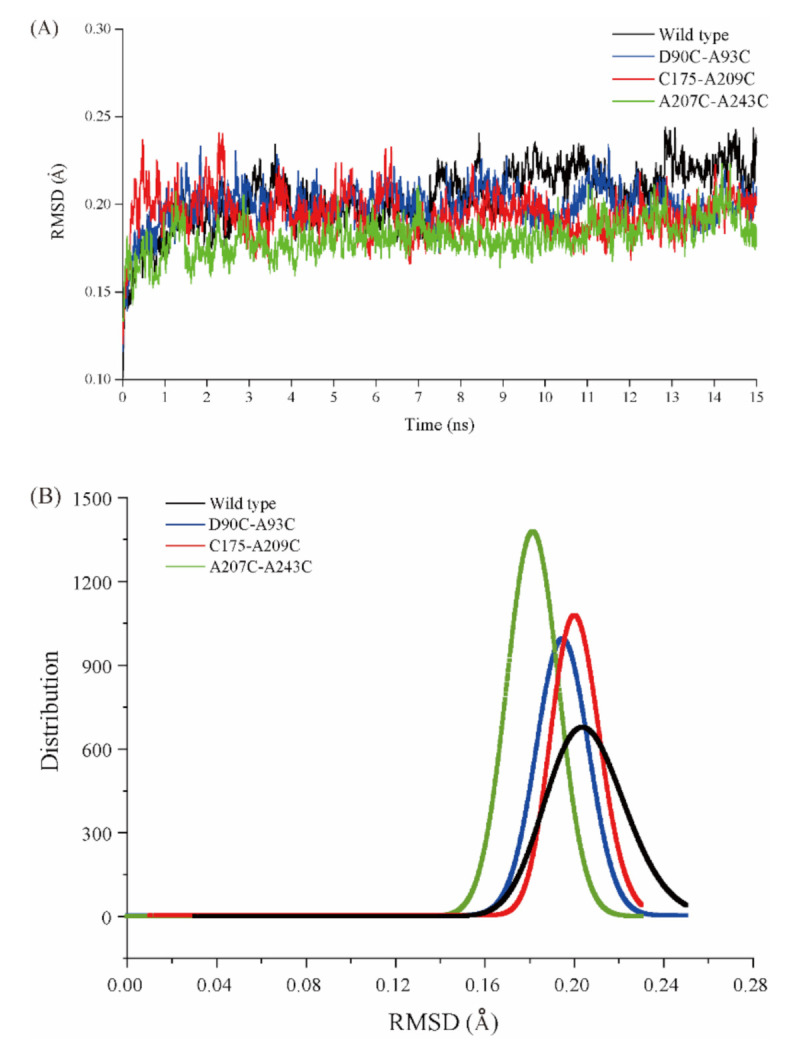

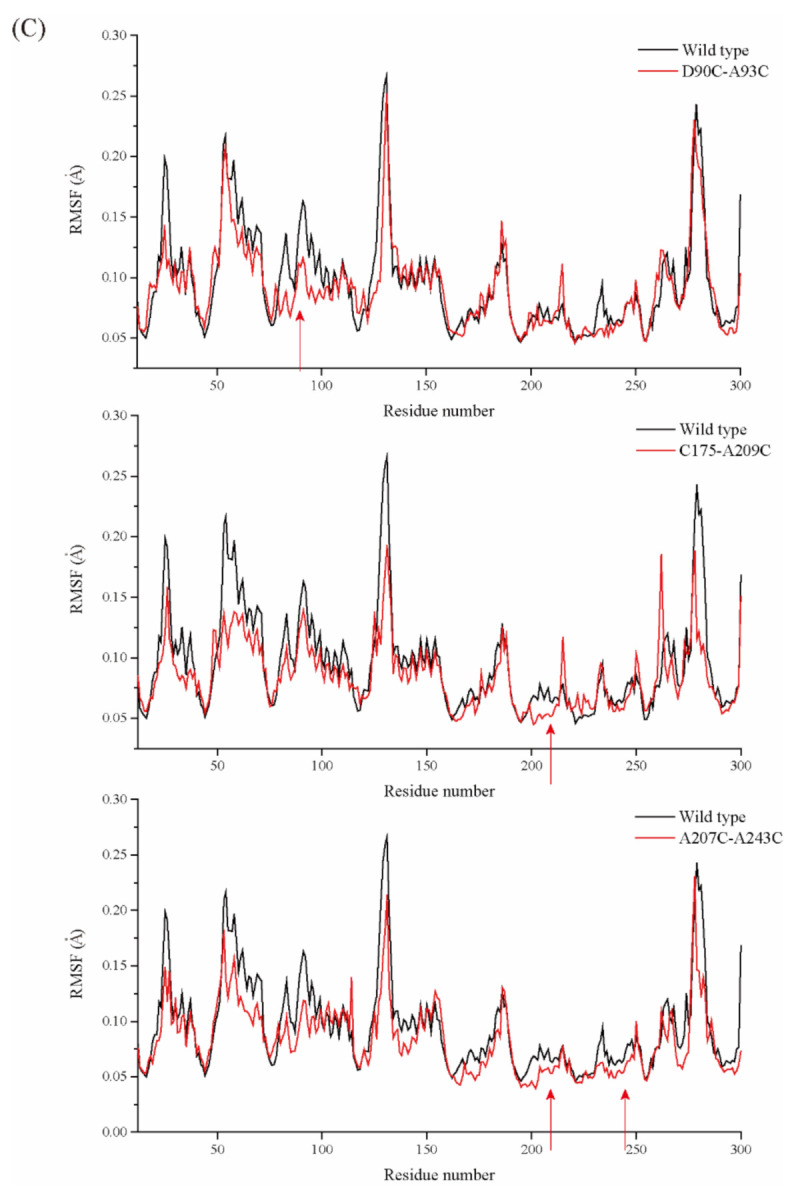
(**A**) RMSD values at 328 K during a 15 ns simulation for wild type DPEase and the three mutants. (**B**) Distribution of the RMSD values. (**C**) RMSF values at 328 K during a 15 ns simulation.

To investigate the changes in the mutants’ structural flexibility at high temperatures, the root mean square deviation (RMSD) and corresponding root mean square fluctuation (RMSF) per residue of the wild-type and the three stabilized mutants were predicted by MD simulation. A low RMSD typically corresponds to high rigidity and potentially high thermostability of the overall structure, and a lower RMSF indicates more rigidity of an individual amino acid residue [33]. At 328 K (55 °C), the MD simulation trajectories of the mutants were almost equal to that of the wild-type, however, the average RMSD was higher for all the residues of wild-type than that of the three mutants (Figure 5A). The RMSD values of Cb-DPEase mainly averaged 0.22 Å while those of D90C-A93C, C175-A209C and A207C-A243C varied around 0.22 Å, 0.20 Å and 0.19 Å, respectively, and the RMSD value distributions greater than 0.23 of the wild type were significantly higher than those of the mutants, indicating that the mutants were much more rigid than Cb-DPEase (Figure 5B). The RMSF values of D90C-A93C, C175-A209C, and A207C-A243C were also markedly reduced at the residue positions (90/93, 175/209, 207/243), where the disulfide bridge was introduced (Figure 5C).

### 2.6. Molecular Modeling of Wild-Type Cb-DPEase and the Mutants

**Figure 6:**
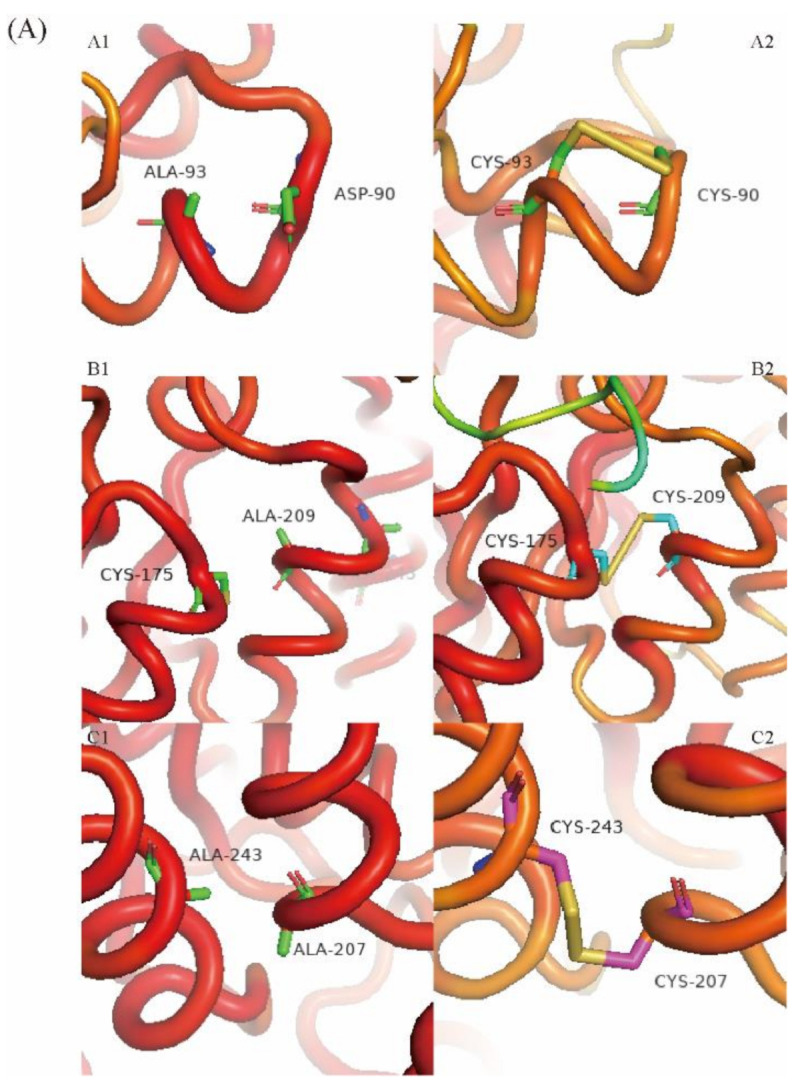

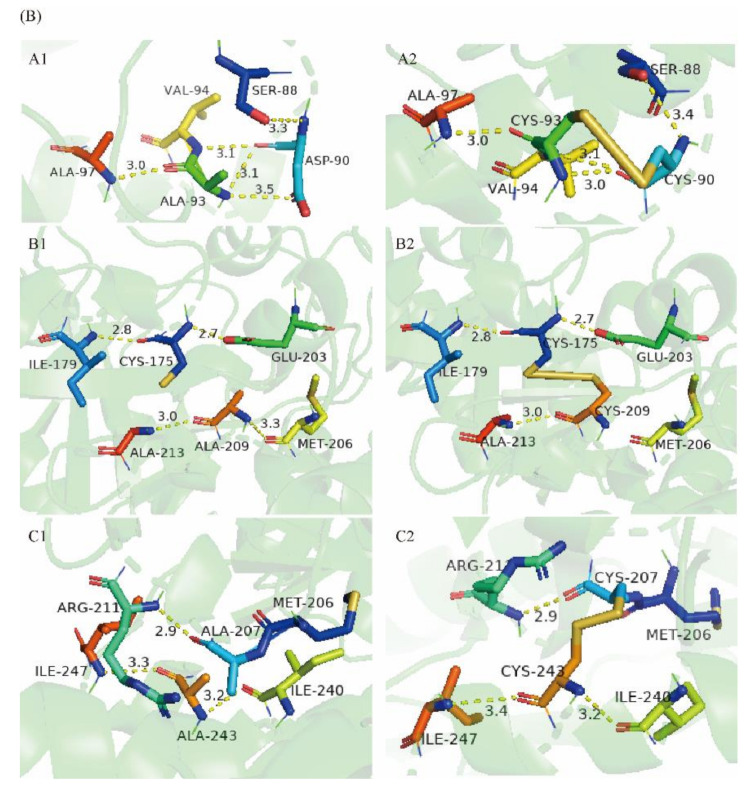
(**A**) 3D Local structures around residues D90/A93 (A1), C175/A209 (B1), and A207/A243 (C1) in wild-type DPEase, with the disulfide bridges in the mutants D90C−A93C (A2), C175−A209C (B2), and A207C−A243C (C2) superimposed. The relative B-factors are shown by color coding; B-factor values are represented by blue, green, yellow, and red in ascending order. (**B**) Local models of 3D structures around residues D90/A93 (A1), C175/A209 (B1) and A207/A243 (C1) residues in wild-type DPEase and the cysteine-substituted mutants D90C−A93C (A2), C175−A209C (B2) and A207C−A243C (C2).

The change of B-factor (a measure of conformational flexibility) around the mutation sites was provided by modeling tools and visualized in Figure 6A. The chain sections around D90/A93, C175/A209 and A207/A243 in the wild-type were relatively flexible, although the flexibility of the loop structure (A1) around D90/A93 was lower than the other two sections. The B-factor around the mutant disulfide bridges in D90C-A93C, C175-A209C, and A207C-A243C had clearly decreased, reflecting the increased conformational stiffness resulting from the introduction of the disulfide bridges.

**Figure A1:**
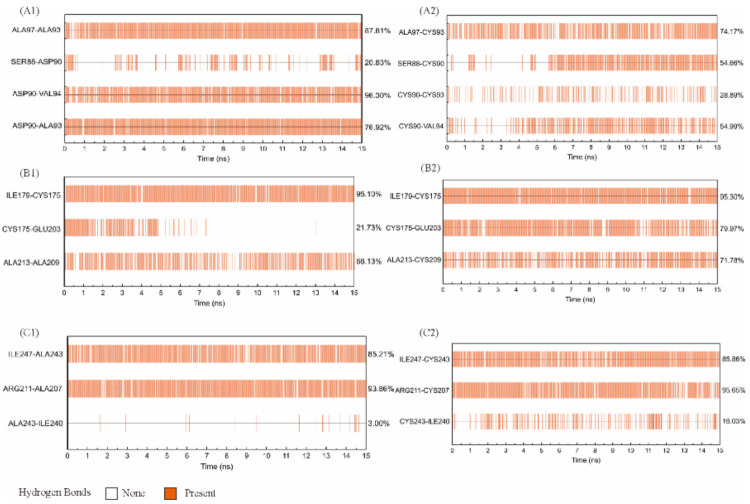
Hydrogen-bond existence map around residues D90/A93 (A1), C175/A209 (B1) and A207/A243 (C1) residues in wild-type DPEase and the cysteine-substituted mutants D90C-A93C (A2), C175-A209C (B2) and A207C-A243C (C2) along the analyzed time frames. Red lines show the presence of a hydrogen bond at that specific time and white breaks imply the absence of that hydrogen bond.

To explore structural changes in the enzyme, induced by the introduction of disulfide bridges, model structures were constructed of Cb-DPEase and its variants, D90C-A93C, C175-A209C, and A207C-A243C. Molecular modeling demonstrated that the new disulfide bridges (Figure 6B), were located between an α-helix and random coil in both D90C-A93C, and C175-A209C, but between two α-helices in A207C-A243C. The hydrogen bond networks were also modified in the 90C-93C and 175C-209C regions. The regions neighboring D90-A93, C175-A209, and A207-A243 in the wild-type contained five, four, and three hydrogen bonds, respectively, whereas one native hydrogen bond (D90-A93/M206-A209) was lost around the mutation sites in both the D90C-A93C and C175-A209C mutants, but the introduction of the disulfide bond did not change the number of hydrogen bonds in the region surrounding A207C-A243C. According to the existence map of hydrogen bonds (Figure A1), these hydrogen bonds (showed in Figure 6B) are statistically present along the MD trajectory. Compared with WT, the stability of hydrogen bonds inside the mutants around D90/A93 was reduced except for SER88-ASP90, but increased around residues C175/A209 and A207/A243. 

## 3. Discussion

Increasing the thermostability of industrial enzymes has always been of central importance to environmentally-friendly industrial production because of the advantages of thermally stable enzymes, such as the lower cost of re-usable enzymes, heat-acceleration of reactions and reduced microbial contamination [34]. Compared with conventional random mutagenesis, disulfide bridge engineering, based on rational design, is a highly-effective method used to generate thermostable mutants of target enzymes [35]. However, incorrect introduction of disulfide bridges may reduce enzyme thermostability [18]. Therefore, greater knowledge about the actual effect of disulfide bridges on the thermostability of DPEase is essential to accelerate the industrial production of D-allulose.

Enzyme denaturation is a two-stage process. The first is a reversible step involving partial unfolding of the peripheral protein chains. The second is exposure of the protein interior, followed by irreversible inter- and intra-molecular aggregation of the hydrophobic regions [36]. Disulfide bridges increase the free-energy barrier of protein unfolding, and thereby reduce the rate of enzyme unfolding, which would account for the observed significant increase in thermostability and optimal temperature (Figure 3A and Figure 4) [37]. Usually, the enzyme optimal pH is determined by the ionizable groups and surface charge of the protein [38]. The introduction of disulfide bridge D90C-A93C shifted the pH optimum from 7.0 to 7.5 (Figure 3B), probably resulting from changes in pKa values of some ionizable groups close to the active site, induced by changes in relative position and non-covalent interactions [39]. Furthermore, the hydrogen bonds lost in the mutated regions may have contributed to the shift in pH optimum, as in a previous report [14].

The commonly accepted prerequisite for high thermostability, i.e., structural rigidity, is higher for thermophilic enzymes than for mesophilic enzymes [40]. The reduction in flexibility of localized regions tends to rigidify the overall protein structure, however, a decrease in local flexibility does not guarantee the rigidification of the overall protein structure, as found for lipase B and glucanase [14,39,41]. Therefore, simultaneously considering changes in localized flexibility, as well as that of the whole protein, is essential for assessing the effects of introduced disulfide bridges. The MD simulation data showed that the addition of each disulfide bridge greatly reduced the flexibility of the nearby protein structure and that of the whole protein, while also enhancing enzyme thermostability (Figure 5A–C). Restricting the motion of the loop/random coil by disulfide cross-linking may have decreased the conformational entropy of the unfolded state (Figure 6A, A1/A2).

In addition to covalent disulfide bonds, hydrogen bonds are undoubtedly the most important non-covalent interactions; the existence of a hydrogen bond network provides a significant contribution to the thermodynamic stability of a protein [42,43]. Changes in the hydrogen bond network were observed around the engineered disulfide bridges; two native hydrogen bonds were lost and five new hydrogen bonds were formed [39]. The introduction of disulfide bridges and the induced new hydrogen bonds can result in rigidification, both locally and overall, therefore, analyzing the spatial configuration of mutants helps to elucidate the molecular mechanism underlying their increased thermostability [41].

According to the modelled 3D structure (Figure 6B), although the number of hydrogen bonds in D90C-A93C and C175-A209C decreased, the disulfide bridges made a major contribution to the increased rigidity of the mutant structures, leading to a significant increase in thermostability. Furthermore, the consolidation of intramolecular hydrogen bonding network in mutants C175-A209C and A207C-A243C actively contributes to the stability enhancement of DPEase (Figure A1), and it might be another reason for the increased thermal stability of the two mutants, in addition to the formation of the disulfide bonds [44].

In conclusion, we evaluated the effect of introducing disulfide bridges on the thermostability of DPEase. The disulfide bridges newly formed by substitution of carefully-selected pairs of amino acid residues by cysteines, appear to decrease both protein flexibility and the entropy of unfolded protein, thereby markedly improving the thermostability of the three mutants. Computational rational design of disulfide bridges combined with MD simulations and spatial configuration analysis provides an effective strategy for modifying the thermostabilities of industrial enzymes.

## 4. Materials and Methods

### 4.1. Gene, Plasmids and Strains

The nucleotide sequence of the DPEase-encoding gene *dpe,* from *Clostridium bolteae* was synthesized with codon optimization by Sangon Biotech Co., Ltd. (Shanghai, China) (Table A1). The *E. coli* strain DH5α and vector pMD19T were used for gene cloning, and the expression plasmid pCold TF that contained *dpe* was used for mutagenesis. The wild-type and mutant DPEs were produced by the *E. coli* strain, Rosetta-gami B (DE3).

### 4.2. Mutagenesis and DNA Manipulations

Site-directed mutagenesis of *dpe* was performed using overlapping extension PCR with pCold TF-*dpe* as the template DNA. The primers used are listed in Table 3. The purified PCR products were ligated into plasmid pCold TF, transformed into *E. coli* DH5α, followed by DNA sequencing and finally transformed into *E. coli* Rosetta-gamiB (DE3) cells.

### 4.3. Production and Purification of DPEase and Its Mutants

A single colony of *E. coli* Rosetta-gamiB (DE3) harboring plasmid pCold TF-Cb or its mutants was inoculated in Luria-Bertani medium (LB, 5 mL) containing ampicillin (100 mg/L) and was cultured at overnight at 37 °C [45]. The seed culture was inoculated at a ratio of 1:100 into LB medium (100 mL) containing ampicillin (100 mg/L) until the OD_600_ reached 0.5–0.6 [46]. Isopropyl *β*-D-1-thiogalactopyranoside (0.25 mmol/L) was added to induce the expression of proteins at 15 °C for 24 h [47]. The crude enzyme was isolated by ultrasonic disruption of the cells (400 w, 2 s pulses, 3 s pauses) for 15 min, then centrifuged at 8000× *g* for 5 min [48]. The target protein was purified by a Ni Sepharose 6 Fast Flow affinity column (the wild-type DPEase and its mutants had been fused with a C-terminal 6-histidine-tag), then dialyzed for 24 h, as described previously [10]. The protein purity and concentration were assessed using sodium dodecyl sulfate-polyacrylamide gel electrophoresis (SDS-PAGE) and the Bradford protein assay, with bovine serum albumin as standard, respectively [49].

### 4.4. Enzyme Activity Assay

The reaction mixture (1 mL) contained purified enzyme (0.5 μmol/L), fructose (50 mg/mL) and Co^2+^ (1 mmol/L) in 50 mmol/L PBS buffer (pH 7.0; Saint Biotech Co., Ltd., Shanghai, China). Reactions were performed at 55 °C for 10 min and were terminated after 10 min by boiling. The resulting D-allulose was measured by high-performance liquid chromatography (HPLC) using a Carbomix Pb-NP column (7.8 × 300 mm, 10 μm, Sepax Technologies, Suzhou, Jiangsu, China) and a 1260 refractive index detector (G1362A, Agilent Technologies Inc., China). The column was eluted at 78 °C with double-distilled water at a flow rate 0.5 mL/min. One unit was defined as producing 1 μmol of newly synthesized D-allulose per minute.

### 4.5. Characterization of DPEase and Its Mutants

The effect of temperature on the enzyme activity was measured over the range of 40–80 °C every 5 °C, at pH 7.0, and the enzyme activities were expressed relative to the highest activity (100%). The thermal stability of the enzyme was determined by incubating the enzyme in sodium phosphate buffer (50 mmol/L, pH 7.0) at 55 °C and samples were taken at appropriate time intervals. The initial activity level before incubation at 55 °C was defined as 100%.

Two buffer systems, sodium phosphate (50 mmol/L, pH 4.5–7.0) and Tris-HCl (50 mmol/L, pH 7.5–8.5), were used to determine the optimum pH of enzyme activity at their optimal temperature. Enzyme activities were expressed relative to the highest activity (100%).

To explore the effects of metal ions, residual enzyme activities were measured in the presence of 1 mmol/L Co^2+^, Mn^2+^, Zn^2+^, Cu^2+^, Ca^2+^, Mg^2+^, or Ba^2+^, at the optimal pH and temperature. The percentage of enzyme activity was calculated relative to the metal ion-free control. All assays were performed in triplicate with three independent measurements.

### 4.6. Structural Modeling

To determine the tertiary structure of *C. bolteae* DPEase, automatic homology modeling was performed via SWISS-MODEL using the Automated Mode method, and the known tertiary structure of *A. tumefaciens* DPEase was chosen as the template. The structural quality control analysis of the final 3D models chosen for Cb-DPEase was carried out using the Structural Analysis and Verification Server tools (SAVES; University of California, Los Angeles, CA, USA) [50].

### 4.7. Verification of Disulfide Bond Formation

The formation of disulfide bonds was determined by quantification of free cysteine in mutant enzymes, using dithionitrobenzoic acid (DTNB) [51]. Enzyme samples of known protein concentration (1 mL) were mixed with DTNB solution (4 mg/mL, 5 mL) and the reaction mixture was incubated at 25 °C for 10 min. The absorbance at 412 nm was measured to assay the cysteine content. The free sulfhydryl group content of the samples was calculated from a standard curve of free cysteine.

### 4.8. Molecular Dynamics (MD) Simulation

The MD simulation was performed with Gromacs 5.04 (Gromacs, Groningen, Netherlands), with the Amberff99SB force field. Each simulation was repeated at least three times from the same initial configurations. Structures were solvated in a cubic box with TIP3P water molecules. Following an energy minimization of 10000 steps by steepest descents, position restrained MD simulation was carried out in an NVT and NPT ensemble over a period of 500 ps, respectively. Then the final production simulations were carried out for a total of 15 ns simulation with a time step of 2 fs at 1 atm pressure and 328 K. VMD and its plugins was employed for the analysis of MD trajectories. The root mean square deviation (RMSD) was calculated for the protein backbone using least-squares fitting, and the root mean square fluctuation (RMSF) was calculated using the coordinates derived from the MD trajectories of the last 5 ns timescale. The distributions of RMSD values were determined statistically with Origin 2018 (OriginLab, Northampton, MA, USA) [52]. The hydrogen bond analysis was performed using VMD [53]. PyMol (PyMOL, Schrödinger, LLC, New York, NY, USA) was used for visualization of the modeled structure [54].

## Figures and Tables

**Table 1:** Cysteine content of wild-type Cb-DPEase and its mutants.

Enzyme	Free Cysteine Concentration (mmol/L)	Free Cysteine Number
Wild type	0.0903 ± 0.02	5
D90C-A93C	0.0874 ± 0.03	5
C175-A209C	0.0720 ± 0.02	4
A207C-A243C	0.0882 ± 0.02	5

**Table 2:** Characterization of the DPEase from different sources ^[a]^.

Sources	Half-Life (*t*_1/2_)	Optimal Metal Ions	Optimal pH	Optimal Temperature	References
*A. tumefaciens*	63.5 min (60 °C)	Mn^2+^	8.0	50 °C	[23]
*Clostridium* sp. BNL1100	15 min (60 °C)	Co^2+^	8.0	65 °C	[24]
*Clostridium cellulolyticu*m H10	9.5 h (55 °C)	Co^2+^	8.0	65 °C	[25]
*C. scindens* ATCC 35704	1.8 h (50 °C)	Mn^2+^	7.5	60 °C	[26]
*Desmospora* sp. 8437	NR	Co^2+^	7.5	60 °C	[27]
*T. primitia*	4 h (50 °C)	Co^2+^	8.0	70 °C	[28]
*Dorea* sp.	NR	Co^2+^	6.0	70 °C	[29]
*Sinorhizobium* sp.	NR	Mn^2+^	8.5	40 °C	[30]
*Arthrobacter globiformis* M30	NR	Mg^2+^	7.5–8.0	70 °C	[31]
*Caballeronia fortuita*	NR	Co^2+^	7.5	65 °C	[32]

^a^ NR: Not reported.

**Table 3:** Oligonucleotides used for the construction of plasmids.

Primer	Sequence ^a^ (5′−3′)
Cb-F	CCGGAATTCATGCGTTACTTCAAAGAAGAAG
Cb-R	CCCAAGCTTAATGGTGATGGTGATGATGACTTGAACCGATACCGAAAACGTGCC
D90/93C-F	GTTCTGAATGTCCTGAATGTGT
D90/93C-R	ACACATTCAGGACATTCAGAACA
A209C-F	GCTGACTGTATCCGTAAAG
A209C-R	CTTTACGGATACAGTCAGC
A207C-F	CAAACATGTGTGACGCTATC
A207C-R	GATAGCGTCACACATGTTTG
A243C-F	GAAATAGGTCAATGTTTACGTG
A243C-R	CACGTAAACATTGACCTATTTC

^a^ NR: The underlined sequences represent the mutation sites.

## Data Availability

The data presented in this study are available on request from the corresponding author.

## Supplementary Materials

The following are available online at https://www.mdpi.com/article/10.3390/ijms221810007/s1.

## Appendix A

**Table A1:** Optimized encoding gene sequences.

**Gene Name**	**Sequence**
Cb-*dpe*	ATGCGTTACTTCAAAGAAGAAGTTGCTGGTATGAAATACGGTATCTA CTTCGCTTACTGGACTAAAGAATGGTTCGCTGATTACAAAAAATACA TGGATAAAGTTTCAGCTCTTGGTTTCGACGTATTAGAAATCTCTTGTG CTGCTTTACGTGATGTTTACACTACTAAAGAACAATTAATCGAATTAC GTGAATACGCTAAAGAAAAAGGTTTAGTTTTAACAGCAGGTTACGGT CCAACTAAAGCTGAAAACCTTTGTTCTGAAGATCCTGAAGCTGTTCG TCGTGCAATGACTTTCTTCAAAGACTTATTACCAAAATTACAATTAAT GGATATCCACATCCTTGGTGGTGGTTTATACTCTTACTGGCCAGTTGA TTTCACAATTAACAACGATAAACAAGGTGATCGTGCTCGTGCAGTAC GTAACCTTCGTGAACTTTCTAAAACTGCTGAAGAATGTGATGTTGTAC TTGGAATGGAAGTTCTTAACCGTTACGAAGGATACATCCTTAACACT TGTGAAGAAGCTATCGATTTCGTTGACGAAATCGGTTCTTCTCATGTA AAAATCATGTTAGACACTTTTCATATGAACATCGAAGAAACAAACAT GGCTGACGCTATCCGTAAAGCTGGTGATAGATTAGGTCATCTTCATTT AGGTGAACAAAATAGATTAGTTCCTGGTAAAGGTAGTTTACCGTGGG CTGAAATAGGTCAAGCATTACGTGATATTAACTACCAAGGTGCTGCT GTAATGGAACCATTCGTAATGCAAGGTGGTACAATCGGTAGTGAGAT TAAAGTATGGCGTGATATGGTTCCAGATTTATCTGAAGAAGCATTAG ATCGTGATGCTAAAGGTGCTCTTGAGTTCTGTAGGCACGTTTTCGGTA TCTAA

**Table A2:** Candidate mutational residue pairs.

Res1 Seq #	Res1 AA	Res2 Seq #	Res2 AA	Sum B-Factors
12	LYS	41	ASP	1.73
17	PHE	31	TYR	1.7
21	THR	26	ALA	1.51
36	SER	70	LYS	1.73
36	SER	72	LEU	1.73
80	PRO	85	ASN	1.54
85	ASN	123	TYR	1.6
86	LEU	144	ASN	1.74
87	CYS	137	ASP	1.72
88	SER	93	ALA	1.74
90	ASP	93	ALA	1.76
109	GLN	155	CYS	1.64
121	TYR	141	ALA	1.75
149	SER	187	SER	1.74
152	ALA	157	VAL	1.71
160	GLY	219	HIS	1.74
164	LEU	169	GLY	1.68
165	ASN	201	ILE	1.69
166	ARG	270	LYS	1.66
167	TYR	167	TYR	1.65
175	CYS	209	ALA	1.76
187	SER	190	VAL	1.75
205	ASN	208	ASP	1.74
207	ALA	243	ALA	1.76
210	ILE	243	ALA	1.75
213	ALA	217	LEU	1.74
224	GLU	228	LEU	1.72
227	ARG	258	PHE	1.71
228	LEU	232	LYS	1.65
229	VAL	291	ALA	1.75
244	LEU	249	TYR	1.72
270	LYS	166	ARG	1.64
279	LEU	283	ALA	1.68
