# Supplementary material for: Enhanced Thermostability of D-Psicose 3-Epimerase from Clostridium bolteae through Rational Design and Engineering of New Disulfide Bridges
Source: Int J Mol Sci. 2021 Sep 16;22(18):10007. doi: 10.3390/ijms221810007 (PMC8464696; doi:10.3390/ijms221810007)
Supplement: Supplementary file 1 [file ijms-22-10007-s001.zip › ijms-1362531-supplementary.pdf]

Supplementary Materials

Enhanced Thermostability of D-Psicose 3-Epimerase from *Clostridium bolteae* through Rational Design and Engineering of New Disulfide Bridges

Jingyi Zhao [1,†] · Jing Chen [1,†] · Huiyi Wang [1] · Yan Guo [1] · Kai Li [1,2] · Jidong Liu [1,2,*]

[1] *College of Light Industry and Food Engineering, Guangxi University, 100 Daxue Road, Nanning 530004, Guangxi, China*

[2] *Sugar Industry Collaborative Innovation Center, Guangxi University, 100 Daxue Road, Nanning 530004, Guangxi, China*

* Corresponding author. Tel: +86 771 3237329. Fax: +86 771 3231590
E-mail: liujd@gxu.edu.cn, liuan6126@126.com

† The first two authors contributed equally to this work.

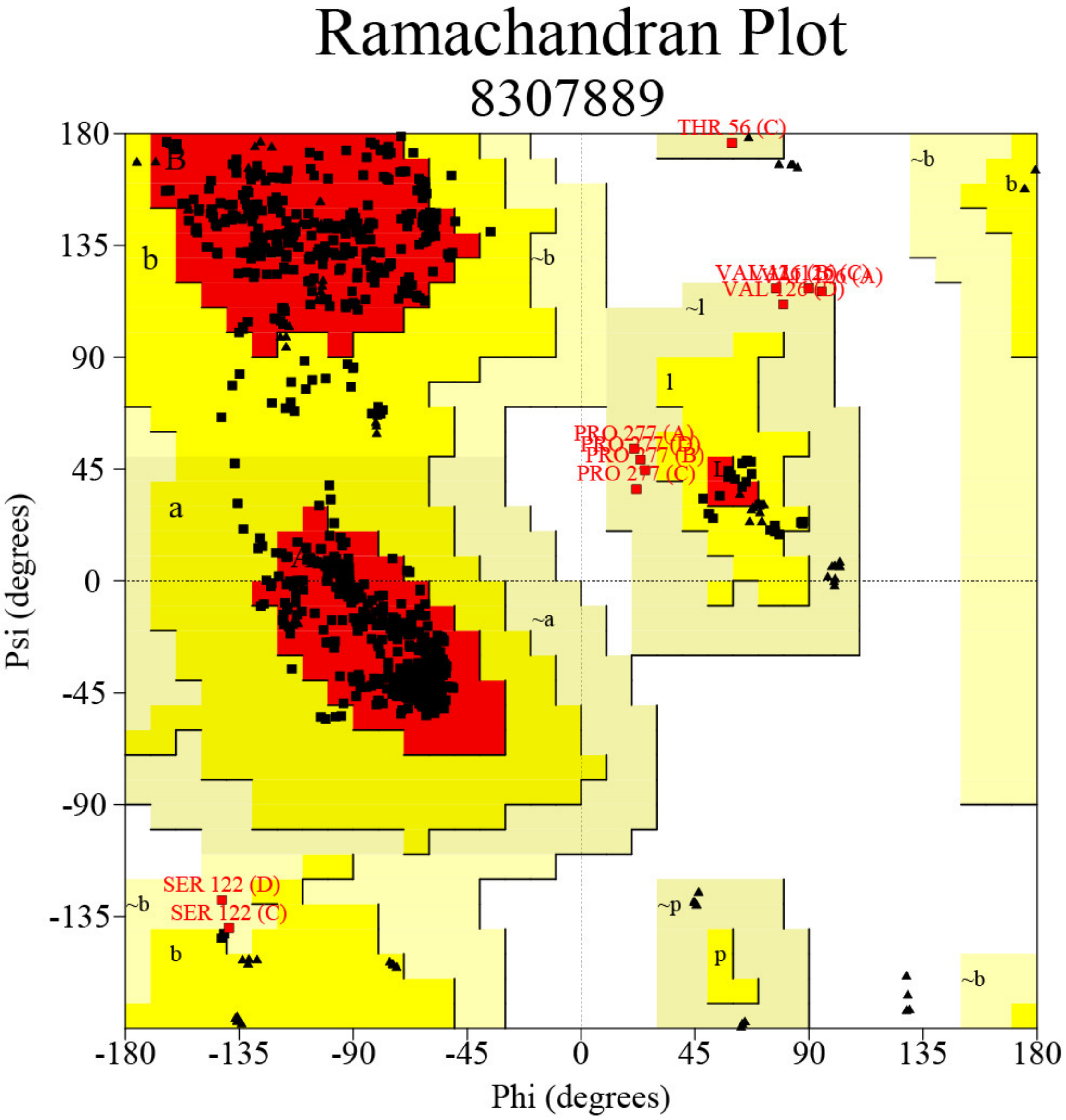

Figure S1. Ramachandran plot of the *C. bolteae* DPEase model.
